# Using the iPod Touch for Patient Health Behavior Assessment and Health Promotion in Primary Care

**DOI:** 10.2196/mhealth.2927

**Published:** 2014-03-21

**Authors:** Samuel N Forjuoh, Marcia G Ory, Suojin Wang, Jude KA des Bordes, Yan Hong

**Affiliations:** ^1^Department of Family & Community MedicineScott & White HealthcareTemple, TXUnited States; ^2^School of Public HealthTexas A&M UniversityCollege Station, TXUnited States; ^3^Department of StatisticsTexas A&M UniversityCollege Station, TXUnited States; ^4^The University of Texas MD Anderson Cancer CenterHouston, TXUnited States

**Keywords:** iPod touch, behavior change, health behavior assessment, health promotion and disease prevention, patient-physician communication, mobile health technology, mHealth

## Abstract

**Background:**

There is a growing recognition of the importance of lifestyle behavior change for health promotion and disease prevention, as well as the concomitant influence of patient–physician communication on effective behavior change. Mobile technology is increasingly being recognized as an important and efficient tool to collect patients’ health behavior data and facilitate patient–physician communication.

**Objective:**

The aim of this study was to assess the feasibility of an iPod touch-based health behavior assessment (HBA) tool in enhancing patient–physician collaborative goal-setting for health promotion in primary care.

**Methods:**

A total of 109 patients from three primary care clinics in central Texas completed a brief HBA, which was programmed on an iPod touch device. An instant feedback report was generated for the patient and their physician simultaneously to facilitate collaborative goal-setting. Within approximately 7 days of the HBA, the patients were phoned for a follow-up survey for their feedback on the iPod touch–based HBA and resultant patient–physician communication.

**Results:**

Patients were able to complete an HBA on the iPod touch with ease. Among those who completed the follow-up survey (n=83), 30% (25/83) reported that their physicians discussed the HBA report with them, while 29% (24/83) established behavior change goals with them. More than 90% (75/83) of the patients reported positive experiences with the iPod touch–based HBA.

**Conclusions:**

It is feasible to use mobile tools for HBA in the primary care setting. The HBA also facilitated patient–physician communication on behavior change. However, more research is needed on the effectiveness of large scale dissemination of mobile-based HBA technology on health communication and behavior change for preventing or managing lifestyle-related chronic conditions, such as obesity, diabetes, cancer, or heart diseases.

## Introduction

Unhealthy behaviors such as physical inactivity, smoking, and unhealthy diets are major contributors to costly chronic health conditions, for instance cancer and other chronic diseases, including diabetes, obesity, and heart diseases [1-3]. For example, despite decades of smoking cessation messages, about 20% of US adults are still current smokers [4]. A large proportion of Americans also exhibit other unhealthy behaviors with less than one-half of adults meeting the US Center for Disease Control’s 2008 physical activity guidelines and more than two-thirds of adults (68.8%) being overweight or obese (body mass index [BMI]>25) [5,6].

Planned health behavior changes are widely recognized as among the most cost-effective interventions for achieving positive health outcomes for prevention and control of chronic diseases [7,8]. For example, one large multicenter trial, the Diabetes Prevention Program, demonstrated that the cost per quality-adjusted life-year was US $1100 for a combined physical activity and nutrition intervention compared with US $31,000 for an intervention based on medication alone [9]. Additionally, the benefits were maintained over an extended period of time [9]. Research also suggests that cueing clinicians to discuss recommendations for behavior change during medical appointments improves physician adherence with health behavior guidelines and clinical outcomes [10]. Once cued, discussions about behavior change can be structured in ways that are most likely to encourage change. For example, smoking patients who were told by their doctors to quit smoking were more likely to take action than their counterparts without physicians’ recommendations [11]. Furthermore, counseling to encourage behavior change may be most effective when the content is individualized to the person for whom the change is recommended [12].

While there are clearly health outcomes and care delivery benefits associated with systematically collecting health behavior data and initiating patient-physician conversation about behavior change, there are practical challenges. Many clinics are understaffed and underresourced and struggle to meet all of the current competing demands they face [13-15]. To meet the growing demands for care, health care systems must maximize the quality and quantity of time spent in patient-physician encounters by integrating innovative technologies to incorporate patient preferences and perspectives, and working within the context of patients’ social networks, cultures, and communities [16].

Interactive behavior change technology (IBCT) has been recognized as a potential resource for improving the effectiveness of health behavior change in health care systems [17]. The past 2 decades have witnessed a rapid development and adoption of mobile health technologies in health promotion and health care [18]. As the penetration of mobile technologies (eg, iPod touch, smart phones, personal digital assistants) in the general population continue to deepen, mobile health technology is increasingly being used as an efficient tool for patient data collection, transport, and storage [19-21]. Additionally, there has been some initial international work in this emerging area of computer-administered health assessments, notably by Goodyear-Smith [22]. If well designed, the application of IBCTs in the primary care setting can be an effective approach to collect health behavior data, facilitate clinician review of patient status, and guide delivery of educational messages and behavioral counseling [18,23,24]. However, there may be potential data privacy issues as well as issues with the accuracy of data with mHealth that must be taken into consideration.

Therefore, the goal of the current study was to assess the feasibility, including usability, acceptability, uptake, and perceived value of using the iPod touch in a health behavior assessment (HBA) in the primary care setting. The reason for choosing the iPod touch was its perceived ease of use, relatively low cost, and adaptability with other mobile devices.

## Methods

### Study Design, Site, and Participants

We conducted a cross-sectional study with data collection at two time periods of approximately 7-days apart in 2011. The study setting was three conveniently selected primary care clinics of a large university-affiliated, multisite specialty health care system in central Texas. The participants were adult patients who had scheduled a routine office visit with their primary care provider in the participating clinics. The criteria for participation included: (1) being at least 18- years old, (2) the ability to speak and read English, (3) having scheduled an appointment for nonurgent care, and (4) arriving at the clinics at least 15 minutes before their scheduled appointments. The study protocol was reviewed and approved by the Scott & White Healthcare institutional review board with no written consent required from subjects.

### Data Collection Procedure

Our information technology specialists programmed the iPod touch device to collect participants’ demographic, general health status, and anthropometric data, along with data on health beliefs and involvement with their health, as well as HBA drawn from North Carolina Health Partners “Starting the Conversation” Series [25] using an interactive, Web-based, touch-screen iPod assessment and report system. A random survey number was generated when the device was handed to each patient. To further protect participant privacy, no data were cached in the iPod touch software so as to prevent going back to prior answers from another patient. The software was tested for usefulness, security, completeness, and stability before being deployed for actual use. The software resided and was run from a Web server that was located in a managed, secure, Intranet data center that used enterprise level back-up and security practices standard for health care and clinical information systems. As an Intranet-based Web solution, the software and data collected were not accessible via the public Internet. Data from the participant responses and generated reports were stored in a secure, relational database that resided in the same data center and was managed using the same health care and clinical information systems practices.

**Figure 1:**
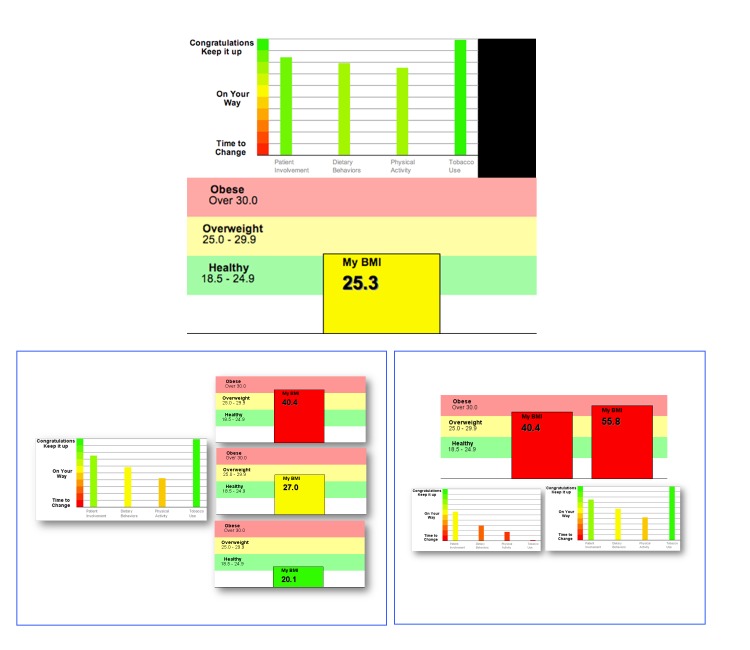
Sample screen shots of iPod Touch HBA.

**Figure 2:**
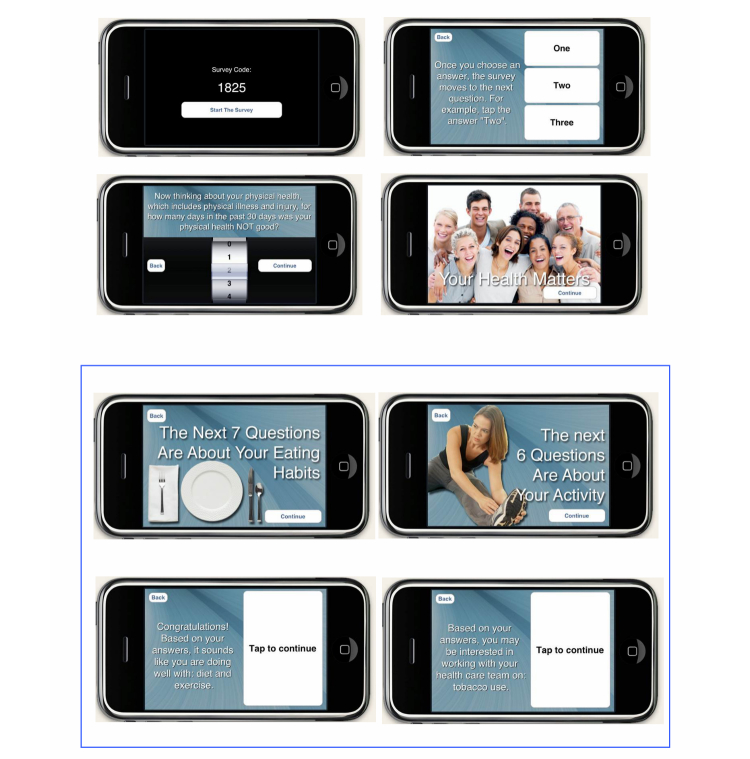
Sample generated HBA summary reports.

Figure 1 displays sample screen shots of the HBA on the iPod touch. After patients checked in and waited for their appointments, a clinical research staff member approached and asked them to participate in the study. After verbal informed consent was given by the patient, the research staff gave them a quick training on how to use and navigate the iPod touch. The training included teaching participants how to make a selection from a choice of answers and how to use the wheel on the device to make a response. After a participant completed the HBA on the secure iPod touch, an HBA summary report was generated and simultaneously sent wirelessly to a research printer (see Figure 2 for sample HBA summary reports). A copy of the HBA summary report was given to the participant and another copy was attached to their folder and given to their physician before encounter with the participant. The physician could use the summary report as a cue to start the conversation and provide appropriate recommendations on behavior change. The participant was provided a monetary compensation of US $20 for their time. Within 7 days of the clinic visit, the patients were contacted again by phone for a follow-up survey regarding their experiences with using the iPod touch and whether they discussed the report with their physician and established any behavior change goals during the clinic visit.

### Measures

We collected the following information using the iPod touch device: (1) demographic data, including gender, age group, and race/ethnicity, (2) health status data, including general health, physical health, and mental health, (3) anthropometric data, including height and weight, (4) data on participant activation or involvement with their own health using 12 questions about health beliefs, such as taking responsibility for their health condition and understanding the nature and causes of their health conditions, and (5) data on health behaviors using seven questions about dietary behaviors, six questions about physical activity behaviors, and six questions about smoking behaviors.

Given our interest in a pragmatic set of measures to assess lifestyle behaviors in primary care settings, we chose to employ questions from the Starting the Conversation Toolkit developed by researchers at the University of North Carolina as a brief tool for rapid lifestyle assessment, behavioral counseling, and  monitoring behavioral change in a variety of health care settings [25].  As indicated in prior work, having a set of brief items is critical for getting assessments integrated into clinical-care settings [26]. Focusing on the behavioral assessment questions from the Starting the Conversation ToolKit, we purposively selected three critical behaviors (tobacco use, eating behaviors, and physical activity) related to the onset and progression of many different chronic diseases [27]. Research in the dietary assessment arena confirms the utility of using this brief screening tool for evaluating intervention impact [28]. These types of Starting the Conversation questions are now part of a harmonized data set for behavioral assessment widely recommended for use in both community and clinical settings [24,29]. Our brief assessment also included items on patient involvement, drawn from the short form of the Patient Activation Measure [30].

Table 1 summarizes the list of the HBA questions. The 12 questions on “participant involvement” required participants to make a choice from a 4-point Likert scale from strongly agree to strongly disagree. The 7 questions on “dietary behavior” required participants to make a choice from three answers (eg, “How many times a week do you eat food that is fried or high in fat?”: 0 = Less than 1; 1 = 1-3; 2 = 4 or more), which were scored 0-2 for a possible total score ranging between 0 and 14, with 0 representing the healthiest eating behavior and 14 representing the unhealthiest. Similarly, physical activity had six questions with a possible score of 0-12 (eg, “How many times a week do you go out for a brisk walk?”: 2 = 4 or more; 1 = 1-3; 0 = Less than 1), with higher mean scores meaning being more active; “tobacco use” also had 6 questions with possible score range of 0-12 with higher mean scores meaning more tobacco use.

The follow-up phone survey comprised 15 items, including 12 questions on their experiences with using the iPod touch on a 5-point Likert scale from strongly agree to strongly disagree, two “Yes-No” questions on their communication with their physicians, and one open-ended question on any other information or feedback they liked to share with us.

**Table 1:** Health behavior assessment questions on the iPod touch.

Patient involvement	Eating behavior	Physical activity	Tobacco use
1. I am responsible for managing my health condition	1. Eat food that is fried or high in fat	1. Go out or a brisk walk per week	1. To perk me up or give me a lift
2. Taking active role in my health is most important factor	2. Servings of fruits or vegetables eaten	2. Hours spent watching TV or on the computer	2. When I am with friends or drinking socially
3. I can take actions to prevent problems with my health	3. Regular sodas and glasses of sweet tea or juice drunk	3. Walk, ride a bike or bus vs. driving	3. Helps me feel comfortable and relaxed
4. I know what my prescribed medications do	4. Eat beans (eg, black beans) chicken or fish	4. Do gardening or intense housework	4. When I’m anxious, worried, depressed or angry
5. I can tell when I need medical care	5. Eat regular (not low-fat) chips or crackers	5. Participate in sports or exercise program per week	5. Within half an hour after I wake up
6. I can tell my provider concerns I have when not asked	6. Eat desserts or other sweets	6. Think of ways to move more vs. less	6. Without really thinking about it
7. I can follow through on medical Rx I need to do at home	7. Margarine, butter, or meat fat use on bread		
8. I understand nature and causes of my health conditions			
9. I know the medical treatment options available			
10. I know how to prevent further problems			
11. I can figure solutions when new problems arise			
12. I can maintain healthy lifestyle changes like diet and exercise			

### Statistical Analysis

Data management, including data entry and coding, recoding, as well as analysis were done using SPSS. As a pilot study focused on feasibility and initial efficacy assessment, data analyses were limited to descriptive statistics. We first computed survey response and follow-up rates. We then performed descriptive statistics on the health behavior measures, participants’ interactions with their physicians, and their experiences and perceptions of the iPod touch device as an assessment tool, including means and standard deviations. Exemplary quotes from participant feedback are included as illustrations.

## Results

### Survey Response and Follow-Up Rates

Of 293 subjects who were approached by the research staff for participation, 27.6% (81/293) were ineligible, 15.4% (45/293) were not interested, and 57.0% (167/293) expressed interest. Of these, 16.2% (27/293) refused verbal consent, while 83.8% (140/293) consented to participate. However, 31 subjects could not complete the HBA survey before being called to the exam room for their physician appointment resulting in a total of 109 subjects who completed the HBA. The mean time needed for participant instruction on navigating the iPod touch device was 43.5 (SD 29.9) seconds.

Of the 109 participants who completed the HBA survey in the clinic, 76.1% (83/109) were successfully contacted for the follow-up phone survey within 7 days of their clinic visit after up to four phone call attempts.

### Subject Characteristics

As shown in Table 2, participants were mostly female (75/109, 68.8%) and non-Hispanic White (69/109, 63.3%), with 15.6% (17/109) being African American and 16.5% (18/109) being Hispanic/Latino. Only 10.1% (11/109) were 65 years or older. The majority was overweight or obese (84/109, 77.1%), while a minority self-reported their general health as fair or poor (25/109, 23.9%). As further depicted in Table 3, the mean BMI of the participants was 30.9 (SD 6.6). Participants also reported approximately 1 week of poor physical or mental health days each month.

Participants generally reported a high involvement with their own health, with the vast majority of them reporting strong agreement or agreement with being responsible for managing their health conditions (107/109, 98.2%), taking an active role in their own health (109/109, 100%), being confident in taking actions that will help prevent or minimize some symptoms (106/109, 97.2%), and having the confidence to tell their health care provider concerns they have when not asked (105/109, 96.3%). While they reported less tobacco use (mean score 1.9, SD 3.7), they reported average behaviors regarding healthy eating (mean score 6.1, SD 2.4) and physical activity (mean score 7.6, SD 2.7).

**Table 2:** Demographics and general health information of participants (n=109).

Characteristic		n (%)
**Age group (years)**		
	18-30	31 (28.4)
	31-45	33 (30.3)
	46-64	34 (31.2)
	64+	11 (10.1)
**Gender**		
	Male	34 (31.2)
	Female	75 (68.8)
**Race/ethnicity**		
	African American	17 (15.6)
	Asian/Pacific Islander	4 (3.7)
	Hispanic/Latino	18 (16.5)
	White	69 (63.3)
	Other	1 (0.9)
**Body mass index (BMI)**		
	Underweight	1 (0.9)
	Normal weight	24 (22.0)
	Overweight	26 (23.9)
	Obese	58 (53.2)
**Self-reported general health**		
	Excellent	6 (5.5)
	Very good	21 (19.3)
	Good	56 (51.4)
	Fair	22 (20.2)
	Poor	4 (3.7)

**Table 3:** Health status and health behavior assessment of participants (n=109).

Characteristic	Mean (SD)
Body mass index	30.9 (6.6)
Number of days physical health not good in the past 1 month	6.3 (8.5)
Number of days mental health not good in the past 1 month	7.1 (8.8)
Number of days unable to do ADLs^a^	5.4 (8.6)
Dietary behavior (0-14)	6.1 (2.4)
Physical activity (0-12)	7.6 (2.7)
Tobacco use (0-12)	1.9 (3.7)

^a^Activities of daily living

### Patient–Physician Communication and Collaborative Goal-Setting

Of the 83 participants who successfully completed the follow-up phone survey, 30% (25/83) reported that their physicians discussed the HBA report with them, while 29% (24/83) established behavior goals related to the HBA report with them.

### Feedback of iPod Touch Device

Table 4 depicts participants’ feedback on using the iPod touch for HBA. Participants generally accepted using iPod touch for HBA, with the vast majority reporting overall positive experiences (82/83, 99%) and acknowledging that the words were easy to see (83/83, 100%), it was easy to use (82/83, 99%), questions were easy to understand (81/83, 98%), they did not believe the iPod touch negatively affected their interaction with their doctor (79/83, 95%), the report was easy to understand (78/83, 94%), and they did not feel rushed to answer the questions (74/83, 89%). Exemplary quotes provided by the participants about their experiences with the iPod touch device corroborated the quantitative findings including comments such as,

Awesome, very easy to read, very quick. Good for people who haven’t used iPod.

Another participant suggested,

I wish they would do everything like that (iPod) because I think it is a lot better than reading and writing. I was able to see the text better on the iPod than on paper.

**Table 4:** Participants’ responses to the follow-up phone survey (n=83)^a^.

Characteristic	Strongly agree/Agree (%)	Not sure (%)	Strongly disagree/Disagree (%)
1. iPod touch device was easy to use	99	1	-
2. Words on the iPod touch easy to see	100	-	-
3. Questions were easy to understand	98	1	1
4. Felt rushed to answer the questions	10	1	89
5. Report was easy to understand	94	4	2
6. Will use information to better health	94	5	1
7. Report makes me want to change	83	11	6
8. Will like to use device more in clinic	94	2	4
9. Report helped to talk to doctor	76	9	15
10. iPod touch negatively affected interaction	4	1	95
11. iPod touch positively contributed to health	86	7	7
12. Overall experience with iPod positive	99	1	-

^a^ *P*<.001 in all cases using the binomial sign test

## Discussion

### Principal Findings

This pilot study assessed the feasibility of using a common mobile tool for HBA and counseling in a primary care setting. Our data suggest that the iPod touch device may be a feasible device to assess lifestyle behaviors. Nearly all participants reported a positive experience overall, finding the words on the iPod touch screen easy to see. The vast majority of patients found the device very easy to use and the questions easy to understand. In addition to its user-friendliness from a participant perspective, as seen in other studies, the iPod touch also offers ease from researchers’ perspective. For example, it minimizes survey response error, it is reliable in eliciting sensitive data in a private and confidential manner, and it is advantageous in terms of easy data storage and transportation.

In addition to being an easy-to-use HBA tool, we also found that the iPod touch was a promising device to assist behavior change in a diverse population of varying age groups, genders, ethnicities, and health status. The generated HBA reports triggered patient-physician collaborative goal-setting for one-third of our patients. This finding corroborates a call to use mobile health technologies or IBCT to promote health behavior change in the primary care setting [18,31].

### Limitations

Several limitations of the study should be noted. First, we had a small and convenient sample recruited from one health care system from one geographical region. Thus, the findings may not be generalizable to other patients in primary care setting. In addition, we did not include children or non-English speaking patients. While promising, further research with a larger sample is needed for better understanding on how different populations may use mobile technology for both HBA and health communication messaging. Second, our follow-up rate was 76.1% (83/109) and we had a short follow-up time (7 days) in the study. Given that this was a pilot study to assess feasibility of using mobile tool for HBA in primary care setting, such a response rate was expected. However, better compliance may have resulted if participants had been compensated after completing all phases of the study instead of only after the iPod touch survey. Further studies are needed to enhance follow-up and better observe how mobile technology might impact long-term behavior change in patients. Third, our study was focused on using the iPod touch device for HBA. Training on how to use the printed HBA reports for better patient–physician communication and goal-setting for behavior change was not included as part of this study. We also did not collect any data on participant technological expertise, or physician’s feedback on using printed HBA reports in their communication with patients, which is needed in future studies. Future research must also include such training for both patients and physicians, so the proportion of patients receiving counseling on behavior change can be increased as well as the assumed effectiveness of such behavioral counseling. Fourth, we used an iPod touch platform for conducting the HBA. In future studies, a comparison of the benefits and costs of iPod touch devices with other mobile devices would be instructive.

### Conclusions

Despite these limitations, the current study suggests that it is highly feasible to use mobile tools for HBA and counseling in primary care settings for the prevention or management of obesity, diabetes, cancer, or heart disease, which are heavily influenced by lifestyle behaviors. Recognizing the magnitude of the prevalence of unhealthy behaviors in Americans, but limited health care resources, Congress passed the Health Information Technology for Economic and Clinical Health Act, which emphasizes the use of electronic health records [32]. Mobile health technologies can be integrated into the increasingly popular electronic health records. Recent studies are also documenting the value of standardizing questionnaires of HBAs and use for patient counseling [24,31]. As the mobile health research accelerates and adoption of electronic health records in health care settings continues, mobile devices such as the iPod touch HBA are promising health communication tools that can be easily integrated into the daily operation of primary care clinics.

## Abbreviations

BMI: body mass index
HBA: health behavior assessment
IBCT: interactive behavior change technology
